# Encapsulation of Grape (*Vitis vinifera* L.) Pomace Polyphenols in Soybean Extract-Based Hydrogel Beads as Carriers of Polyphenols and pH-Monitoring Devices

**DOI:** 10.3390/gels8110734

**Published:** 2022-11-11

**Authors:** Gianluca Viscusi, Elena Lamberti, Carmela Gerardi, Giovanna Giovinazzo, Giuliana Gorrasi

**Affiliations:** 1 Department of Industrial Engineering, University of Salerno, Via Giovanni Paolo II, 84084 Fisciano, Italy; 2 National Research Council-Institute of Science of Food Production (CNR-ISPA), Via Monteroni, 73100 Lecce, Italy

**Keywords:** soybean extract, ionotropic gelation, wine pomace, anthocyanin, pH sensitivity, hydrogels, grape pomace extract

## Abstract

In this work, novel bio-based hydrogel beads were fabricated by using soybean extract as raw waste material loaded with Lambrusco extract, an Italian grape cultivar. The phenolic profile and the total amount of anthocyanins from the Lambrusco extract were evaluated before encapsulating it in soybean extract-based hydrogels produced through an ionotropic gelation technique. The physical properties of the produced hydrogel beads were then studied in terms of their morphological and spectroscopic properties. Swelling degree was evaluated in media with different pH levels. The release kinetics of Lambrusco extract were then studied over time as a function of pH of the release medium, corroborating that the acidity/basicity could affect the release rate of encapsulated molecules, as well as their counter-diffusion. The pH-sensitive properties of wine extract were studied through UV-Vis spectroscopy while the colorimetric responses of loaded hydrogel beads were investigated in acidic and basic solutions. Finally, in the framework of circular economy and sustainability, the obtained data open routes to the design and fabrication of active materials as pH-indicator devices from food industry by-products.

## 1. Introduction

Anthocyanins are a class of water-soluble polyphenol pigment derived from fruits and vegetables [1]. They are classified as the most important pigments of vascular plants and are responsible for the typical colors of vegetables and fruits [2]. From a chemical point of view, they are glycoside and acylglycoside derivatives of six common anthocyanidins—cyanidin, delphinidin, malvidin, pelargonidin, peonidin and petunidin—which are oxygenated heterocycles with two aromatic rings connected by three carbons [3]. These are classified according to the number and position of hydroxyl groups on the flavan nucleus [4]. Meanwhile, anthocyanins exhibit antioxidant and antibacterial activity and are suitable for incorporation into films to develop bioactive packaging [5]. Notably, anthocyanins change their structures and colors as pH varies [6]. Thus, anthocyanin-rich films are considered potential intelligent and natural pH indicators to monitor the freshness of food since it is known that there is a relationship between the pH value of foods and their spoilage [7]. Some examples regard the fabrication of anthocyanin-loaded agar/potato starch [8]; cellulose nanofiber packaging film based on purple sweet potato anthocyanin and oregano essential oil [9]; anthocyanin-associated purple sweet potato starch and peel-based pH-indicator films [10]; *Echium amoenum* anthocyanins loaded into bacterial cellulose film [11]; anthocyanin-loaded gelatin films [12]; and anthocyanin-loaded ovalbumin-propylene glycol alginate nanocomplexes [13]. However, the stabilization and delivery of these molecules is challenging since they are unstable under certain environmental conditions, resulting in low bioavailability. To overcome this, different strategies to encapsulate hydrophilic anthocyanin-based extracts have already been explored to preserve their characteristics and reduce their degradation [14,15]. In order to protect the active compound, an encapsulation process was considered and applied. The fundamental goal is to improve the protection of the core material from external factors such as light, moisture and oxygen [16]. To fulfill this, ionic gelation can be applied as a microencapsulation methodology that may be performed through simple procedures. This technique appears to be simple, low-cost and mild, and thus, is useful for encapsulating substances that are easily degraded [17]. Particulate forms of gel present some useful applications due to their capacity for absorbing water and biological fluids. Moreover, they are able to adapt in shape and size, enabling controlled release of the active compounds in agricultural, pharmaceuticals or food products [18,19]. By definition, hydrogels are classified as polymeric networks with hydrophilic properties known as “ionotropic hydrogel”, produced by combining a polyelectrolyte with a multivalent ion with an opposite charge [9]. Ionic gelation allows for the production of a porous gel matrix without using high temperatures and organic solvents [14]. The methodology was applied to efficiently encapsulate reactive compounds, such as anthocyanins and phenolic compounds [20]. Biodegradable polymeric matrices such as cellulose, agarose, starch, alginate, carrageenan, gum arabic, chitosan, gelatin and albumin are commonly employed [21,22,23,24,25]. Moreover, alternative food chain wastes can even be used to produce bio-based carriers for the delivery of active compounds. In this study, soybean extract was obtained from aqueous soy seeds extract. It is a liquid mixture rich in protein and carbohydrate which was used for the fabrication of polymeric hydrogel carriers [26,27]. To facilitate the gelation process, sodium alginate was used as a gelling agent. Alginate is a natural polysaccharide derived from brown seaweed [28]. When coupled with whey protein isolate and other materials, sodium alginate enhances the encapsulation efficiency of encapsulated compounds [29]. In contact with divalent cations, such as Ca^2+^, alginate can form ionic reversible hydrogels via intermolecular crosslinking [30,31,32]. The interactions with blocks of G monomers in alginate result in ionic bridges between different polymer chains, known as the “egg-box model” [33], leading to the stabilization of the network and the formation of stable gel capsules [13]. So, the ionic gelation methodology was applied to the design of novel hydrogel systems based on soybean aqueous extracts and sodium alginate to incapsulate freeze-dried polyphenol aqueous extract obtained from the pomace of Lambrusco grape, a typical Italian grape variety. Current scientific research is focusing on the selection of natural colorants and biopolymers for the fabrication of pH-responsive indicators due to their non-toxic, safe and easily degradable properties [34]. To the best of the authors’ knowledge, there have been no available scientific studies regarding the formulated biocomposites to date.

## 2. Results and Discussion

### 2.1. Lambrusco Pomace Extract Characterization

With the aim of producing a functional extract using grape pomace, we optimized the extraction of different classes of polyphenols with biological activity in water acidified with citric acid. The preliminary results suggest that 5% grape pomace powder in water is the percentage at which the saturation of polyphenol extractability is reached. In this aqueous extract, we characterized different groups of phenols, flavanols (catechin and epicatechin), flavonols (quercetin, rutin, quercetin 3-glucosilate and kaempferol 3-glucosilate), soluble acids (gallic acid, caftaric acid and cutaric acid) and anthocyanins. The results of the GP phenolic profile, antioxidant activity (AA) and total phenolic (TP) content are reported in Table 1 and Table 2. Anthocyanins expressed as oenin (mg/g) and flavanols (Catechin and Epicatechin) were found to be the most representative constituents. All these compounds and their antioxidant activity characterize the grape pomace as a raw material rich in bioactive compounds with antioxidant and anti-inflammatory activity.

### 2.2. Morphological Analysis of Beads

**Figure 1:**
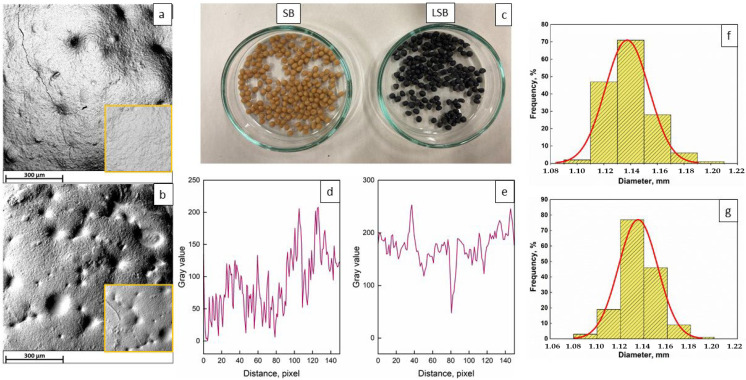
SEM micrographs of SBs (**a**) and LSBs (**b**). Appearance of SB and LSB (**c**). Surface plot profiles for SBs (**d**) and LSBs (**e**). Diameter distributions of SBs (**f**) and LSBs (**g**).

Figure 1 shows SEM micrographs of soy extract-based beads.

From the SEM micrographs, it is evident that both the specimens show the formation of a surface texture with regular forms and quite homogeneous and even surfaces for SBs, while some pores and combes can be observed after the introduction of grape pomace extract. However, the surface morphology does not seem to greatly change after the introduction of GP extract. No cracks or wrinkles are present. The profiles of the surfaces show some differences between the two samples. The SBs appear to be rougher than LSBs, as demonstrated by the highly scattered profiles, while the LSB surfaces are more homogeneous with some sharp peaks belonging to the combe-like formation observable in the SEM micrograph. This result is confirmed by the evaluation of R_a_ and RMS parameters: The SBs possess an R_a_ of 152.0 and an RMS of 170.6, while the LSBs show an R_a_ of 113.9 and an RMS of 137.1.

**Figure 2:**
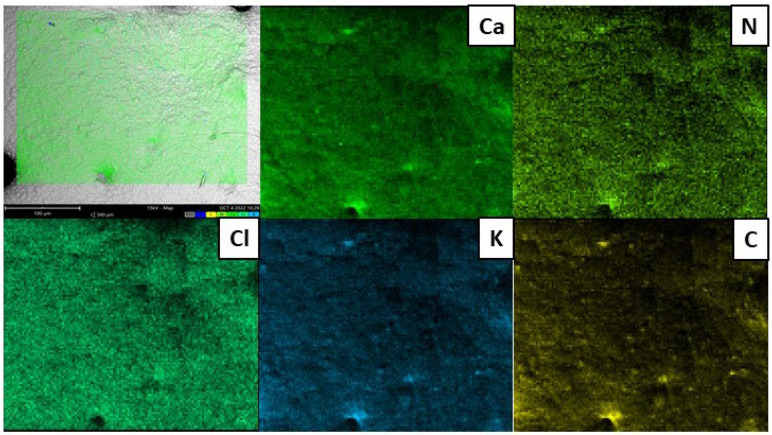
EDX maps of Lambrusco polyphenol-loaded beads (LSBs).

EDX spectra (Figure 2) demonstrated the presence of characteristic elements such as calcium, nitrogen, chloride and potassium. The nitrogen presence could be related to the high protein content of soybean extract. Conversely, calcium and sodium could be associated with CaCl_2_ and sodium alginate residues. Finally, chloride’s presence could be associated with malvidin-3-glucoside, delfinidin-3-glucoside, cyanidin-3-glucoside and peonidin-3-glucoside. The elemental mappings confirm that these elements are homogeneously distributed on the surface.

### 2.3. FTIR Analysis

**Figure 3:**
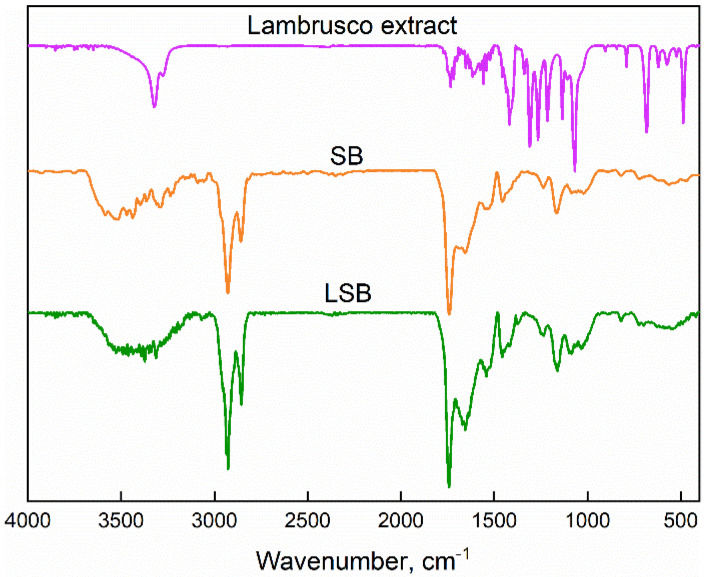
FTIR spectra of Lambrusco extract and soybean extract-based beads.

Figure 3 shows the FTIR spectra of the Lambrusco extract, the pure soybean extract-based bead and the encapsulated Lambrusco one.

The FTIR spectrum of Lambrusco extract evidences peaks of carbohydrates, identified by the bands located at 816 cm^−1^ (α–glycosidic C-H bonds), 1200–1300 cm^−1^ (stretching of the pyran ring) and 1440 cm^−1^ (aromatic rings) [35]. The absorption band at 3320 cm^−1^ is related to the stretching of -OH groups in phenolic compounds and water, while the band at 1265 cm^−1^ concerns the C-O groups in the pyran rings from flavonoids [36]. Other signals include those at 1617 cm^−1^ (C=O), 1041 cm^−1^ (C-O) [37], 675 cm^−1^ (C-H aromatic) and 1260 and 1076 cm^−1^, related to the skeletal stretching vibration of aromatic rings and the C-O-C group in flavonoids [38]. The soybean extract-based bead shows a peak located at about 3443 cm^−1^ (elongation of the OH groups of polymeric compounds such as alcohols and carboxylic acids), 2921 cm^−1^ (CH symmetric and asymmetric vibration and aliphatic acid elongation) and 1720 cm^−1^ (asymmetric stretching vibration of C=O). Other peaks are located at 1666, 1538 and 1233 cm^−1^ (amide I (C=O stretching vibration), amide II (N-H bending vibration) and amide III (C-N and N-H stretching vibrations) of the proteins, respectively) [39]. The encapsulated LSBs show characteristic bands of both Lambrusco extract and SBs.

### 2.4. Swelling Degree Analysis

**Figure 4:**
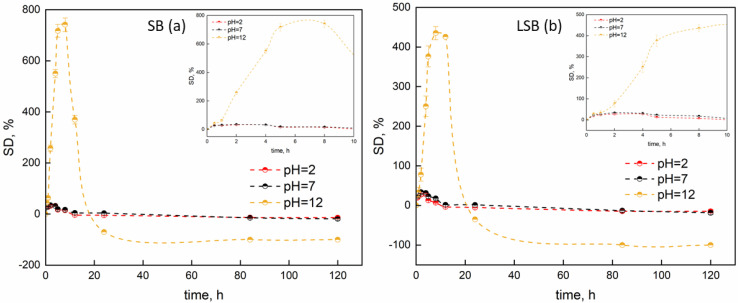
Swelling data for SBs (**a**) and LSBs (**b**) as function of pH level.

Figure 4 reports the swelling behavior of SBs and LSBs at the three tested pH levels.

In acidic and neutral media, no noticeable differences could be observed. Regarding pH = 2, SBs absorbed water up to SD = 32% while LSBs underwent a swelling phenomenon up to 28% after 4 h. After that, the erosion and dissolution phenomena led to the dissolution of the polymeric matrix and a deswelling phenomenon was observed, with a decrease in weight of up to 14%, which appeared to be quite stable over time. So, the presence of Lambrusco extract did not noticeably affect the swelling behavior of soybean extract-based beads. In neutral conditions (pH = 7), swelling up to 34% and 32% for SBs and LSBs was verified before they underwent deswelling. Finally, in basic conditions (pH = 12), a completely different phenomenon was observed. For short times, the rate of water uptake sharply increased, and then, leveled off. As pH increased, -COOH groups were transformed into -COO- groups, leading to the breakage of hydrogen bonds, while the acidic amino acids (Glu, pK_a_ = 4.25; Asp, pK_a_ = 3.86) were also completely ionized. Thus, the electrostatic repulsions within the test hydrogel beads make the hydrogel swell more. For example, alginate beads tend to swell when placed in basic pH solutions since the alginate molecules are highly charged and tend to repel each other [40,41]. The presence of hydrostatic repulsions between macromolecules is due to water molecules, which lead to expansion of the polymeric structure [42] and an increase in mean diameter over time. Regarding the presence of Lambrusco powder, the loading seemed to reduce the increase in volume and the swelling phenomena of beads. This was justified by the fact that the main components of Lambrusco extract are highly polar, so they can expose a high number of polar sites; these could interact with the polar sites of soybean extract, contributing to the formation of an interconnected network. After a certain time, dissolution phenomena occur and deswelling is verified. For long durations (about 8 h), the swelling ratio decreases evidently due to the dissolution of sodium alginate and the rupture of the composite hydrogel beads. In highly alkaline conditions, the hydrostatic repulsion between charged anionic groups can induce the collapse in the structure, leading to deswelling phenomena.

### 2.5. Release Kinetics of Encapsulate Hydrogels Beads

**Figure 5:**
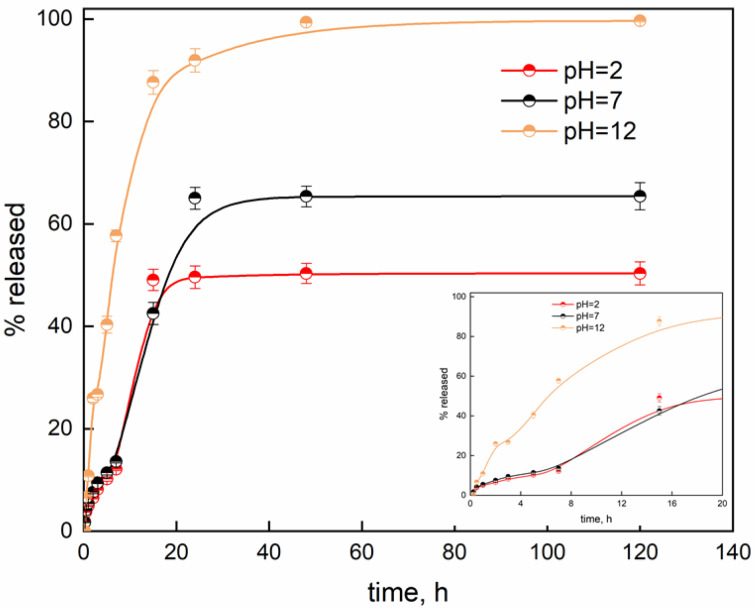
Release kinetics of Lambrusco polyphenol-loaded soybean-based beads.

To test the capability of soybean extract-based beads to be used as controlled release systems for the delivery of polyphenols, release kinetics were evaluated using a UV-Vis spectrophotometer. Their release from the beads was followed over time and is reported in Figure 5 as a function of pH.

**Figure 6:**
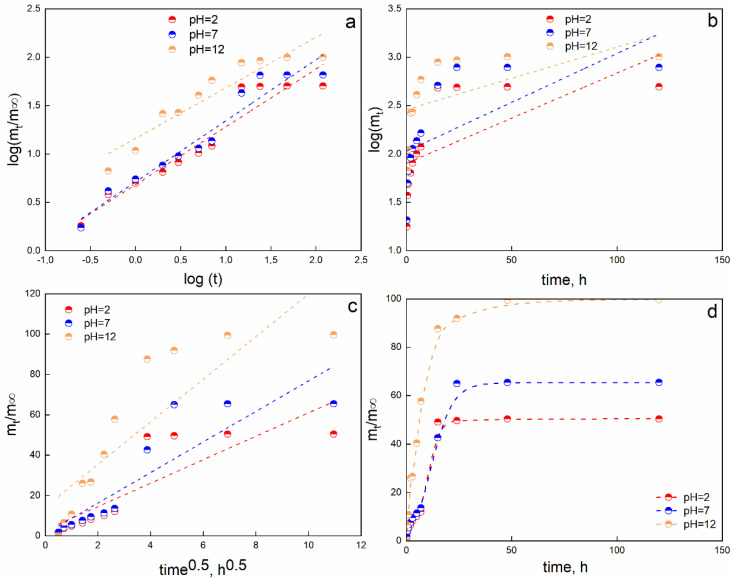
Release data fitted through Korsmeyer–Peppas (**a**), first-order (**b**), Higuchi (**c**) and modified Weibull (**d**) models.

The release of an encapsulated substance can be considered a gathering of complex phenomena which are correlated to different factors, such as the physicochemical properties of the polymer and the solute, the structural characteristic of the polymeric system, the release environment, etc. The release of the grape pomace extract could be associated with the entry of water molecules from the liquid medium. The counter-diffusion movement of polyphenol molecules to the external surface follows. In general, the mechanism of release of encapsulated molecules is known to be due to many parameters (physical properties, porosity, drug-to-polymer ratio, specific area, pH, and swelling and deswelling phenomena) [43]. Figure 6 shows that the release rate of GP extract is dependent on the pH of the liquid medium. The initial low burst release for pH = 2 (10%) and pH = 7 (11%) is supposed to be related to the free functional substances in compliance with the slow increase in volume due to the swelling phenomenon, as previously reported. After 7 h, an evident increase in the released amount is observed before reaching a plateau regime after 24 h and releasing 50% at pH = 2 and 65% at pH = 7. The release in the pH = 12 medium is noticeably different. Chain relaxation and gel expansion are due to increased electrostatic repulsions generated between the negative charges of the carboxylic groups. So, a high burst release was observed with about a 60% release after 7 h. Moreover, the release occurring at pH = 12 allowed the total amount of released polyphenols to level off after 48 h. This phenomenon could be related to the relaxation of polymer chains and hydration due to the penetrating water molecules [44]. The adsorbed water molecules could then increase the internal pressure, allowing the GP extract to be released with a burst release effect. As pH is reduced, the soybean-based hydrogel structure appears to be more stable, and the beads are able to adsorb fewer water molecules, resulting in an increase in swelling. The better stability at low pH could determine mass transfer resistance for the counter-diffusion of GP extract; it could slow down the release rate, justifying the lower amount released. The release kinetics were fitted through the previously reported models (Figure 6).

As clearly evidenced in Figure 6, the considered models are not able to fit the release data, apart from the modified Weibull model (R^2^ = 0.9994). So, the release profiles could be studied by considering a two-mechanism process: diffusion and relaxation. The parameters of interest obtained from the fitting process are reported in Table 3.

The parameter θ accounts for the diffusion phenomenon that occurred at relatively short durations. At high pH levels, relaxation phenomena occurred; the counter-diffusion of Lambrusco extract could mainly be due to the opening of the polymeric structure as a result of the high amount of water that entered due to the swelling process. The 1/A_1_ and 1/A_2_ ratios could be associated with the kinetic constants of the diffusion and relaxation phenomena, respectively. So, the kinetic constants of pH = 2 and pH = 7 are quite similar, while the 1/A_1_ ratio for pH = 12 is quite low, proving that the main mechanism of release is related to relaxation phenomena instead of diffusion ones (97.8%). Additionally, the 1/A_2_ ratio shows a opposite trend, with a higher release constant for release in basic medium. This statement is even confirmed by the highly negative value of b_1_ for pH = 12, which contributes to making the diffusion mechanism quite negligible. Additionally, it is worth noting that in neutral and acidic medium, a quasi-double-step process can be observed with a t_m_ of 5.2 h and 4.2 h for pH = 2 and pH = 7, respectively. The more controlled release rate of GP at pH = 2 and pH = 7 could be due to the high stability of soybean extract-based hydrogels and the lower swelling degree. The interconnected network could then act as a mass transfer barrier, which induces slowing of the release rate. The second release step contribution (42%, 51% and 97.8%) is characterized by a faster relaxation-controlled mechanism (high power of parameter b). Finally, a plateau regime is reached.

### 2.6. Colorimetric Response of Lambrusco Extract Solutions

**Figure 7:**
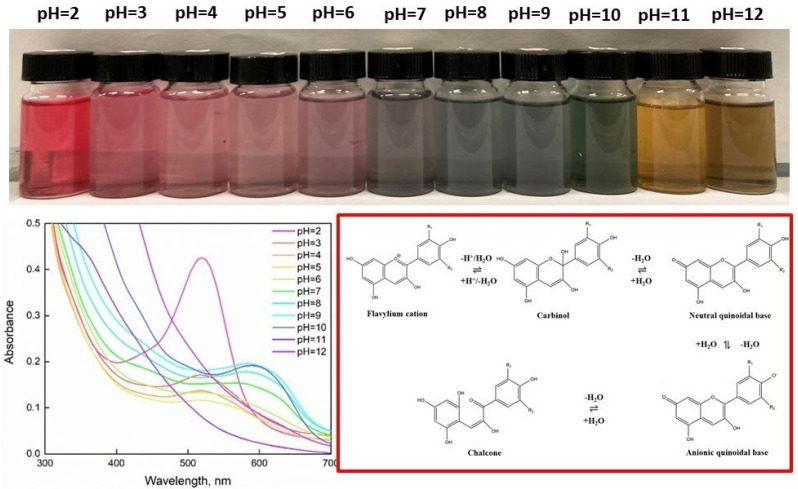
Color variations of Lambrusco GP-derived anthocyanins in different buffer solutions of different pH (2–12).

Figure 7 shows the colorimetric responses of anthocyanins of Lambrusco solutions in different buffer solutions (pH = 2–12).

**Figure 8:**
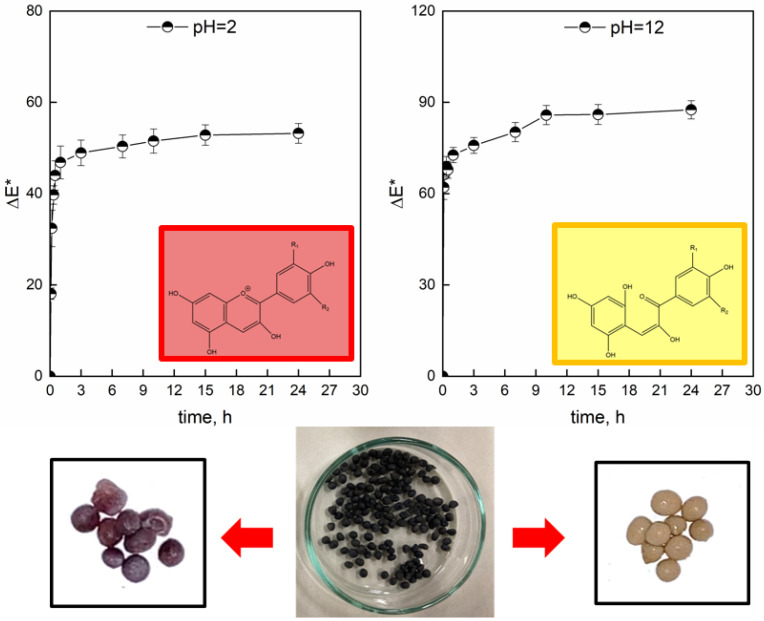
Colorimetric responses of LSBs at pH = 2 and pH = 12. Below: pictures of LSBs in different buffer pH solutions after 24 h.

As shown above, the color of the solution appears red at pH < 4, light red at pH = 5–6, light blue at pH = 7–8, blue-green at pH = 9, and shifts to yellow at pH > 10 [45]. At pH = 2, a maximum absorbance was reached at 520 nm. A bathochromic shift effect was verified with an increase in pH values, displacing the peak from 520 nm at pH = 2 to higher wavelengths up to 610 nm at pH = 12. The deprotonation of OH groups at low pH led to the formation of the quinonoid bases, which resulted in a chromatic shift toward higher wavelengths. At a higher pH, further deprotonation generated an anionic quinoidal base, inducing a red shift in the adsorption spectrum. These changes in color are ascribed to structural changes in anthocyanins at different pH levels [46], mainly by cyanidin-3,5-O-diglycoside, which can interact in different ways. The equilibrium structures are: red flavylium cation (pH < 4), colorless carbinol pseudobase (pH = 4–5), blue/purple quinonoidal anhydrobase (pH = 6–8) and light yellow or colorless chalcone (pH > 8) [47]. Structural transformations of anthocyanins on the basis of different pH are reported in Figure 8 [48]. In this way, Lambrusco extract showed potential for application as a pH-sensitive device.

### 2.7. Colorimetric Response of Lambrusco Extract-Loaded Hydrogel Beads

Figure 8 represents the variation in ΔE* over time, obtained through values of the CIE L*a*b coordinates. Pictures of the color response behavior of the colorimetric hydrogel beads to pH = 2 and pH = 12 solutions are even reported.

The LSBs were kept in contact with buffer solution at pH = 2 (H^+^ = 0.01 mol/L) and pH = 12 (OH^−^ = 0.01 mol/L). The change in L*, a* and b* proves that the color of the beads is affected by the pH of the medium. The trends of ΔE* are well-described by a power law with R^2^ = 0.9956 (Equations (1) and (2)):

(1) $$∆E^{*}\left(\mathrm{pH}=2\right)=\frac{732.5*t^{1.3}}{1+14.32*t^{1.3}}$$

(2) $$∆E^{*}\left(\mathrm{pH}=12\right)=\frac{175.5*t^{0.17}}{1+1.47*t^{0.17}}$$

**Figure 9:**
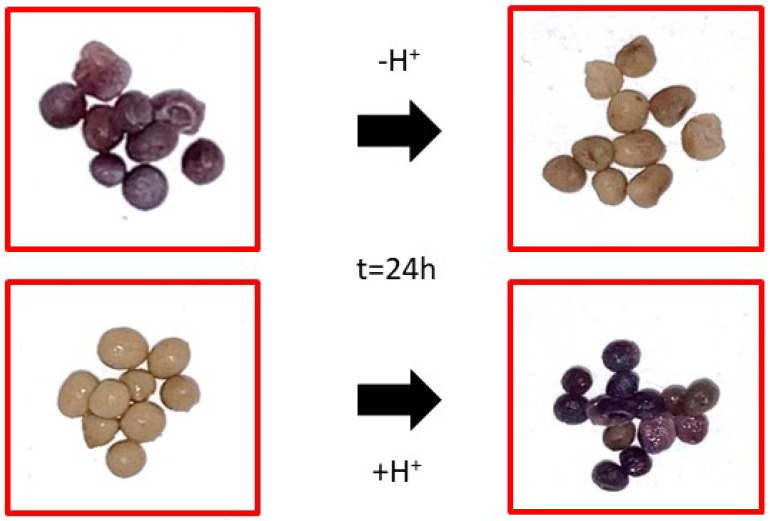
Reversibility of Lambrusco anthocyanin-encapsulated beads.

The equations reported above show that, in basic buffer solution, the rate of increase is higher compared to pH = 2. The contact with H^+^ ions and OH^−^ ions determined structural changes in the anthocyanins, resulting in color changes. The a* and b* color coordinates varied as a function of time and H^+^ concentration. As expected, the a* value became higher when the beads were left in media with lower pH (pH = 2), showing a red color due to the dominance of the flavylium cation (a* = 57.36 and b* = 2.84 after 24 h). By increasing the pH, OH^−^ causes changes in anthocyanin structures [49]. So, the a* values decreased to −6.07 (a* < 0 indicates a green color) while the b* value increased to 26.87, resulting in a color tendency to olive-yellow, which was caused by the yellow chalcone. As can be seen, the ΔE* values sharply increased for both tests, but the sensitivity to the basic solutions of the hydrogels beads was always higher compared to pH = 2. These results are consistent with the photographs of the hydrogel beads after the color changes. The ΔE* values were at a maximum after about 10 h. The obtained results suggest that hydrogel beads can have the role of visual pH sensors. After 24 h, the smart hydrogel beads were removed from the pH = 2 and pH = 12 buffer solutions, wiped and placed into pH = 12 and pH = 2 fresh buffer solutions, respectively. The main idea was to verify the reversibility of the colorimetric response and the practical application of LSBs as sensing devices (Figure 9).

According to Figure 9, the red beads (pH = 2) changed to olive-yellow in an alkaline environment. Furthermore, the olive-yellow beads changed to red-crimson after contact with acid solution for about 1 h. Therefore, the developed systems have good reversibility of color change, which could be applied in smart sensor applications. Generally, color changes can be detected with the human eye when ΔE is higher than 5. Therefore, all the color changes in LSBs can be detected with the naked eye [50]. The use of extract from wine grape pomaces opens a route to the fabrication of novel carriers, potentially usable for food packaging applications, as delivery systems for polyphenols or as pH-monitoring devices, according to the statements of sustainability and circular economy.

## 3. Conclusions

In this study, aqueous extracts from soybean were used to produce novel bead hydrogel loaded with grape pomace extract, a by-product of Lambrusco Italian wine. Alginate as a forming agent was used and the beads were produced through gelation in CaCl_2_ solution. SEM micrographs highlighted the roughness and the presence of combes on the surface of the hydrogels beads. The presence of the main elements (Ca, N, K, Cl) was confirmed by EDX maps. Swelling phenomena were investigated over time (up to 120 h) upon varying the pH of the media. The release kinetics of GP appeared to be affected by pH. Finally, the colorimetric response of hydrogel beads was investigated through evaluation of CIE L*a*b parameters in pH = 2 and pH = 12 buffer solution. The reversibility of the colorimetric response was even investigated. From the obtained data, it can be stated that a novel green delivery system and pH-monitoring device has been designed to achieve controlled release of a grape pomace extract and to detect pH variations.

## 4. Materials and Methods

### 4.1. Materials

Sodium alginate (SA) (CAS: 9005-38-3), calcium chloride (CaCl_2_) (CAS: 10043-52-4), 37% *v*/*v* hydrochloric acid solution (CAS: 7647-01-0) and NaOH in pellet form (CAS: 1310-73-2) were purchased from Sigma-Aldrich (St. Louis, MO, USA). Sodium chloride (CAS: 7647-14-5) was purchased from Carlo Erba Reagents (Cornaredo, Italy). Authentic standards of kuromanin (cyanidin 3-O-glucoside chloride), oenin, quercetin, rutin (quercetin 3-O-rutinoside) kaempferol, catechin and epicatechin were purchased from Extrasynthèse, Genay, France. Gallic acid, coutaric acid, caftaric acid, Folin–Ciocalteu phenol reagent, Trolox [(S)-(-)-6-hydroxy-2,5,7,8 tetramethylchroman-2-carboxylic acid], ABTS [2,2′-azino-bis (3-ethylbenzothiazoline-6-sulfonic acid)], acetonitrile, ethanol, formic acid and organic acids (all HPLC grade) were purchased from Sigma-Aldrich, St. Louis, MO, USA. In all experiments, Milli-Q (Merck Millipore, Darmstadt, Germany) water was used. Soy extract (SE) was obtained from soy seeds purchased from a local supermarket.

### 4.2. Lambrusco Pomace Powder Extraction

Pomace (GP) from the grape cultivar Lambrusco (*Vitis vinifera* L.) was obtained from a local winery. GP was dried in an oven at 50 °C, until a constant weight (48 h in the dark) [51]. With the aim to produce a green extract using GP, we optimized the extraction of different classes of polyphenols with functional activity in water acidified with citric acid. GP extracts were obtained via infusion of 5% (*w*/*v*) GP, 1% (*v*/*v*) citric acid 1 M in water at 80 °C for 5 min. The GP extract was dried using a Freezone^®^2.5 model 76530 lyophilizer (Labconco Corp., Kansas City, MO, USA) until a constant weight.

### 4.3. Bead Preparation

**Figure 10:**
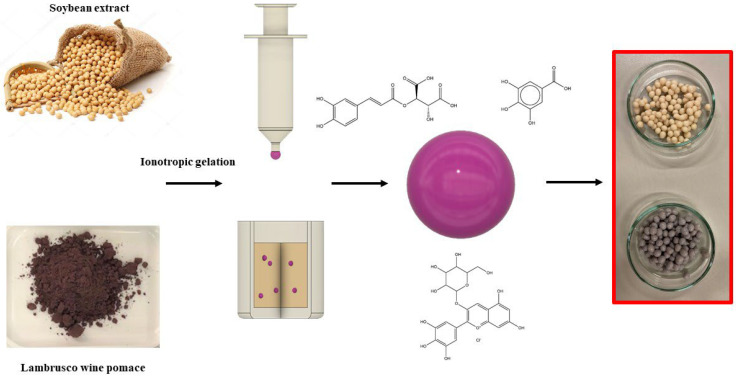
Schematization of ionotropic gelation and production of soybean extract-based beads.

Soy seeds were gently washed three times before soaking in distilled water (1:4 *w*/*w*) for 24 h. Then, soaked soy seeds were ground to obtain soy paste and soybean extract (SE) was obtained by squeezing the paste through a nylon cloth. Sodium alginate (0.4 g) was added to 15 mL of soybean extract solution as a gelling agent. The mixture was dissolved and stirred at 60 °C for 3 h. The solution was then cooled to 40 °C and 0.2 g of Lambrusco extract was added. After complete solubilization, the solution was added dropwise to 5% wt CaCl_2_ solution and stored at 4 °C for 24 h. The beads were recovered from the CaCl_2_ solution and washed with distilled water. Finally, the produced beads were air-dried for 72 h. Hereafter, the Lambrusco-loaded beads will be referred to as LSBs. Sodium alginate/soybean extract beads (SBs) were fabricated according to the same procedure. Figure 10 schematizes the gelation process and, by way of example, shows a picture of the produced beads.

### 4.4. High-Performance Liquid Chromatography (HPLC) Characterization of Anthocyanins

In order to carry out quantification and characterization of the anthocyanin molecules in the GP extract, HPLC analysis (Agilent-1100 liquid chromatograph equipped with a DAD detector) was performed according to the procedure reported by Gerardi et al. (2020) [36]. The column used was a C18 Luna (Phenomenex, 250 × 46 mm, 5 μm) in conjunction with a C18 guard cartridge column (T = 30 °C). The mobile phase was as follows: (A) water/formic acid = 95/5 and (B) acetonitrile/formic acid = 95/5. The samples were eluted following a linear gradient: 1 min of isocratic elution with 6.7% B, 25 min of linear gradient from 6.7 to 16.7% B, 9 min of linear gradient from 16.7 to 55.6% B, 5 min of isocratic elution with 55.6% B, 3 min of linear gradient from 55.6 B to 80% B, and 8 min of isocratic elution with 80% B. An equilibration time of 10 min was set before the next injection while the flow rate was set at 0.7 mL/min. Chromatograms were acquired at 520 nm. Total anthocyanins were directly quantified via the HPLC/DAD (R^2^ ≥ 0.99) setting as reference compounds the malvidin 3-O-glucoside (oenin) and expressed as oenin equivalents (OEqs).

### 4.5. HPLC Characterization of Phenolic Acids, Stilbenes, Flavanols and Flavonols

RP-HPLC DAD (Agilent 1100 HPLC system, Santa Clara, CA, USA) was applied to separate the phenolic compounds in the GP extract according to the methodology described by Calabriso et al. (2022) [52]. This was carried out on a C18 column (5 UltraSphere, 80A pore, 25 mm), with a linear gradient from 20% to 60% acetonitrile, for 55 min (solvent A consisted of 1% H_3_PO_4_ while solvent B was made of 100% acetonitrile) with a fixed flow of 1 mL/min at 25 °C. The chromatographic analysis was carried out by comparing the results with the retention time of standard compounds.

### 4.6. Trolox Equivalent Antioxidant Capacity (TEAC) Assay

The assay was performed as previously described with minor modifications, as reported in Gerardi et al. (2020) [53].

### 4.7. Folin–Ciocalteu Assay

The total phenol in water extracts from dried grape pomace was evaluated using a rapid microplate methodology [54]. The assay was carried out in a 96-well plate (Corning) using a microplate reader (Tecan, Infinite M200). The gallic acid standard solution or sample (50 μL) and Folin–Ciocalteu Reagent (FCR) (1:5, *v*/*v*, 50 μL) were put in each well and 100 μL of NaOH solution (0.35 M) was then introduced. After a duration of 5 min, Abs = 760 nm was evaluated. Regarding the sample absorption, 50 μL of 0.4 M acetic acid solution was used instead of Folin–Ciocalteu Reagent. The reagent blank was evaluated via the addition of 50 μL of H_2_O instead of a standard compound or sample. The experiments were completed in triplicate at T = 25 °C. The calibration curve of gallic acid was found to be in the range of 2.5–40.0 mg/L with a coefficient of determination of R^2^ ≥ 0.999. The absorbance values obtained for samples were related to those of the gallic acid standard curve.

### 4.8. pH Sensitivity of GP Extract

The pH-responsive characteristics of GP extract were determined by adding 0.2 mL GP solution (0.5 g/L) into buffer solution (pH = 2–12). The spectra of the samples were recorded using a spectrophotometer in the range of 400–700 nm.

### 4.9. Characterization of Hydrogel Beads

SEM analysis was conducted to investigate the morphology of the beads. Images were acquired using a Phenom ProX microscope in high-vacuum mode. An integrated energy-dispersive X-ray diffraction (EDS) detector was used for elemental analysis. The diameter of the beads was evaluated by analyzing digital images and they are expressed as the mean ± standard deviation.

Plot profiles were obtained from SEM images through the Plot Profile plug-in in Fiji software. Image processing analysis allowed us to display a two-dimensional graph of the intensities of pixels along a line within the image by selecting an area of roughly 1000 μm^2^. The *x*-axis represents the distance along the line and the *y*-axis is the pixel intensity. Finally, surface roughness parameters were evaluated though mathematical equations. R_a_ (arithmetic average of the absolute values of the profile height deviations from the mean line within the evaluation length) and RMS (root mean square average of the profile height deviations from the mean line within the evaluation length (L)) were evaluated using Equations (3) and (4):

(3) $$R_{a}=\frac{1}{L}*\int_{0}^{L}\left|Z\left(x\right)\right|\mathrm{dx}$$

(4) $$\mathrm{RMS}={\left[\frac{1}{L}*\int_{0}^{L}Z{\left(x\right)}^{2}\mathrm{dx}\right]}^{1/2}$$

where Z(x) is the profile height function. The analysis of plot profiles was repeated in triplicate.

Fourier transform infrared (FT-IR) absorption analysis was performed using a Bruker spectrometer (Bruker Italia, Milano, Italy), model Vertex 70 (average of 64 scans, at a resolution of 4 cm^−1^).

Swelling properties were determined as described hereinafter. A total of 10 mg of beads were air-dried and weighed before we immersed them in a determined volume of liquid (15 mL). After determination of the intervals, wet beads were wiped in order to remove excess liquid. After that, the weights were recorded. The effect of pH on swelling degree was studied by testing hydrochloric acid (1 M) and sodium hydroxide (1 M) solutions. The swelling degree (SD) was calculated using Equation (5):

(5) $$\mathrm{SD}\left(\%\right)=\frac{W_{w}-W_{\mathrm{dry}}}{W_{\mathrm{dry}}}\;\times \;100$$

where W_w_ and W_dry_ refer to the weights of the specimens measured at a determined time point and the initial weight, respectively. The evaluation of swelling properties was repeated in triplicate.

The release profiles of anthocyanins were performed in triplicate by using an ultraviolet spectrometric measurement (Spectrometer UV-2401 PC Shimadzu (Japan)). A determined number of beads were immersed in 30 mL of liquid at a fixed stirring rate of 300 rpm in an orbital shaker (VDRL MOD. 711+ Asal S.r.l.). At determined time intervals, 3 mL of release medium was withdrawn and the absorbance of the band at 258 nm was recorded. The experimental release points were fitted with the following models: the modified Weibull model (Equation (6)), first-order model (Equation (7)), Higuchi model (Equation (8)) and Korsmeyer–Peppas model (Equation (9)) [55].

(6) $$\frac{M_{t}}{M_{\infty }}=\theta^{*}\left[1-\exp(-\frac{t^{b_{1}}}{A_{1}})\right]+(1-\theta {)}^{*}\left[1-\exp(-\frac{{\left(t-t_{m}\right)}^{b_{2}}}{A_{2}})\right]$$

(7) $${\log\;M}_{t}={\log\;M}_{t,0}+\frac{k*t}{2.303}$$

(8) $$\frac{M_{t}}{M_{\infty }}{=k}_{H}*t^{0.5}$$

(9) $$\frac{M_{t}}{M_{\infty }}{=k}_{R}*t^{n}$$

where M_t_ is the amount of substance released at time t, M_∞_ is the amount of substance released at infinite time, M_t,0_ is the initial substance amount, T represents the latency time, the scale factor A_i_ accounts for the time dependence, and b_i_ is related to the substance-release mechanism. k_1_, k_H_ and k_R_ are the respective release constants. Moreover, n represents the exponent of diffusion [56]. The first term of the modified Weibull model concerns the diffusion-controlled mechanism, while the second one is associated with the relaxation phenomena.

Color difference (ΔE) values were measured using ColorimeterX software. The color difference calculation was performed based on the CIE L*a*b color difference equation [57] (Equation (10)):

(10) $$∆E^{*}=\sqrt{{\left(L_{2}{}^{*}-L_{1}{}^{*}\right)}^{2}+{\left(a_{2}{}^{*}-a_{1}{}^{*}\right)}^{2}+{\left(b_{2}{}^{*}-b_{1}{}^{*}\right)}^{2}}$$

L^∗^ characterizes the grey value and it ranges between 100 (white) and 0 (black), while a^∗^ and b^∗^ are the chromaticity coordinates. Subscript 1 refers to the state before UV exposure, while subscript 2 denotes the state after UV exposure. The (a_2_^∗^ − a_1_^∗^) positive values describe a red shift; (a_2_^∗^ − a_1_^∗^) negative values describe a green shift, (b_2_^∗^ − b_1_^∗^) positive values characterize a yellow shift, and (b_2_^∗^ − b_1_^∗^) negative values refer to a blue shift [58]. Each sample was measured four times and the data are reported as the average values with standard deviations. The color parameters, such as the L*, a*, b* values of beads, were measured using ColorimeterX software. The analysis of color differences was carried out in quintuplicate.

### 4.10. Statistical Analysis

Statistical Analysis was carried out through an ANOVA test coupled with Tukey’s post hoc method, with the *p*-value set at lower than 0.05. Statistix 8.1 software (Analytical Software, Miller Landing Rd, Tallahassee, FL, USA) was used to perform the analysis.

## Figures and Tables

**Table 1:** Characterization of the phenolic profile of a lyophilized water extract of total Lambrusco pomace.

Sample	Gallic Ac	Catechin	Epicatechin	Quercetin	Quercetin-3 Gluc mg/g	Rutin	Kaempferol-3-Gluc	Caftaric Ac	Cutaric Ac
Lambrusco	0.62 ± 0.02	1.32 ± 0.13	2.04 ± 0.05	0.15 ± 0.01	0.900 ± 0.03	0.202 ± 0.03	0.091 ± 0.04	0.228 ± 0.08	0.051 ± 0.02

**Table 2:** Characterization of total anthocyanin content (Tot. Ant.) expressed as oenin equivalents (OE), main non-acylated anthocyanins, antioxidant activity (AA) and total phenol content (TP) in lyophilized water extract of Lambrusco pomace.

Sample	Tot. Ant. mgOE/g	Malvidin-3-Gluc mg/g	Delfinidin-3-Gluc mg/g	Cyanidin-3-Gluc mg/g	Peonidin-3-Gluc mg/g	AA mmolTE/g	TP mgGAE/g
Lambrusco	31.52 ± 1.20	12.291 ± 0.49	12.141 ± 0.49	0.166 ± 0.06	1.458 ± 0.08	1.415 ± 0.07	62.842 ± 7.28

**Table 3:** Modified Weibull model’s parameters.

Sample	θ	A_1_ (h^b1^)	A_2_ (h^b2^)	b_1_	b_2_	t_m_ (h)
pH = 2	58	20.5	35.2	0.49	1.27	5.2
pH = 7	49	22.1	43.2	0.47	1.46	4.2
pH = 12	2.2	4.6 × 10^14^	9.15	−102.5	1.08	0

## Data Availability

Not applicable.
